# Understanding vitiligo-related concerns through online discourse: an AI-assisted cross-platform study of Chinese and global communities

**DOI:** 10.3389/fpubh.2026.1832699

**Published:** 2026-05-13

**Authors:** Hongjie Luo, Xinjin Liu, Yukun Wang, Xian Jiang

**Affiliations:** 1 Department of Dermatology and Venerology, West China Hospital, Sichuan University, Chengdu, China; 2 Laboratory of Dermatology, Clinical Institute of Inflammation and Immunology, Frontiers Science Center for Disease-related Molecular Network, West China Hospital, Sichuan University, Chengdu, China

**Keywords:** artificial intelligence, public health, self-reported concerns, social media, vitiligo

## Abstract

**Objective:**

To examine major vitiligo-related concerns expressed in Chinese and international social media discourse, and to compare differences in high-frequency questions across two online platforms.

**Methods:**

This study analyzed publicly available vitiligo-related posts from Baidu and Reddit published between October 2021 and October 2025. After stratified random sampling by publication year and quality screening, 2,305 posts were included (Baidu: *n* = 1,414; Reddit: *n* = 891). An AI-assisted workflow was used for translation, keyword extraction, and standardization of user-expressed questions, followed by researcher-led validation and thematic categorization. Extracted content was grouped into nine thematic categories. Cross-platform differences in high-frequency standardized questions were assessed using two-proportion z-tests with Benjamini–Hochberg correction for multiple comparisons.

**Results:**

A total of 6,375 keywords were extracted and normalized into 2,419 unique terms. Intervention and treatment were the most frequent thematic category (36.3%), followed by diagnosis and detection (20.9%), psychological impact and progression (10.0%), and hereditary and pediatric factors (7.7%). Across platforms, treatment-related concerns were the dominant shared topic. Compared with Reddit, Baidu showed higher proportions of posts related to healthcare provider or hospital selection, treatment prognosis and recurrence, psychological stress, treatment considerations for special populations, vaccination or preventive measures, and disease transmissibility. In contrast, Reddit showed higher proportions of posts related to medication side effects, treatment accessibility, dosage and treatment duration, and interpretation of diagnostic tests. In addition to treatment and diagnosis, online discussions also highlighted psychosocial distress, stigma, family-related concerns, and relationship anxiety.

**Conclusion:**

Vitiligo-related social media discussions on Baidu and Reddit were dominated by treatment-related concerns but differed substantially in the distribution of high-frequency questions across platforms. These findings suggest that social media may provide a useful supplementary window into unmet informational, emotional, and practical needs related to vitiligo, and may help inform more responsive patient education, digital health communication, and patient-centered care strategies.

## 1 Introduction

Vitiligo is a common acquired depigmentation disorder characterized by well-demarcated milky-white or porcelain-white patches. These patches can affect any body surface, with a predilection for exposed and friction-prone areas, significantly affecting patients’ physical appearance and social functioning (1, 2). As a global disorder, vitiligo affects approximately 0.06–2.28% of the population without gender differences (3). Although the age range is broad, the disease predominantly affects young individuals, with nearly half of cases developing before the age of 20 (4). Vitiligo occurs across all ethnic groups, and the contrast between depigmented lesions and normal skin is often more pronounced in individuals with darker skin phototypes, which may intensify its psychosocial impact and negatively affect quality of life and treatment experiences (3–5).

The pathogenesis of vitiligo is multifactorial and remains incompletely understood (6, 7). In addition to autoimmune and oxidative mechanisms, increasing evidence suggests that psychological stress may interact with disease activity through the neuro-immuno-cutaneous axis, linking emotional burden with disease perception and progression (8, 9).

Given this prominent psycho-dermatological interplay, patients with vitiligo frequently experience intractable psychological distress (4, 6, 7, 10, 11). The chronic progressive nature of vitiligo, combined with visible disfigurement and societal stigmatization, contributes to persistent shame, social avoidance, and diminished self-esteem. Consequently, anxiety and depression prevalence rates in vitiligo patients significantly exceed, with this burden being particularly pronounced among female and younger patients (12, 13).

In recent years, social media has become an increasingly important space in which health-related concerns are expressed, negotiated, and shared (14–16). Individuals living with chronic and visible skin conditions often turn to online platforms to seek information, share treatment experiences, express emotional distress, and discuss challenges that may not be fully captured in conventional clinical encounters (17). Social media therefore provides a valuable window into concerns related to vitiligo in real-world settings. However, existing evidence in this field has largely relied on questionnaire-based investigations, clinical observations, or analyses restricted to single platforms and relatively small samples. As a result, large-scale and naturally occurring vitiligo-related discussions across different sociocultural contexts remain insufficiently explored (18, 19).

In this study, we aimed to identify the major concerns and unmet needs of vitiligo through naturally occurring discussions on social media. To enable a cross-cultural comparison of online discourse perspectives, we focused on two widely used platforms representing different online communication environments. Baidu is one of the most widely used online information and discussion ecosystems in China and serves as an important channel through which Chinese users search for health information, raise disease-related questions, and engage in public discussions (18–21). Reddit, in contrast, is a widely used English-language discussion platform that facilitate relatively open and peer-driven exchanges about health experiences (22, 23). Despite differences in language and broader context, posts on both platforms typically follow similar structures, including problem description, question framing, and peer response. This structural similarity allows for comparative analysis of user-expressed concerns across platforms.

Given the relative large volume of unstructured user-generated content, we employed an AI-assisted analytical approach between September and November 2025 using GPT-5 (OpenAI, default setting, temperature: 0.6), an advanced large language model that has increasingly been explored for information extraction, text analysis, and knowledge synthesis across multiple domains and digital platforms (24). By comparing vitiligo-related discussions across these two platforms, this study sought to provide evidence that may support more responsive patient education, improve health communication, and inform patient-centered management strategies for vitiligo.

## 2 Materials and methods

### 2.1 Study design and data collection

**Figure 1:**
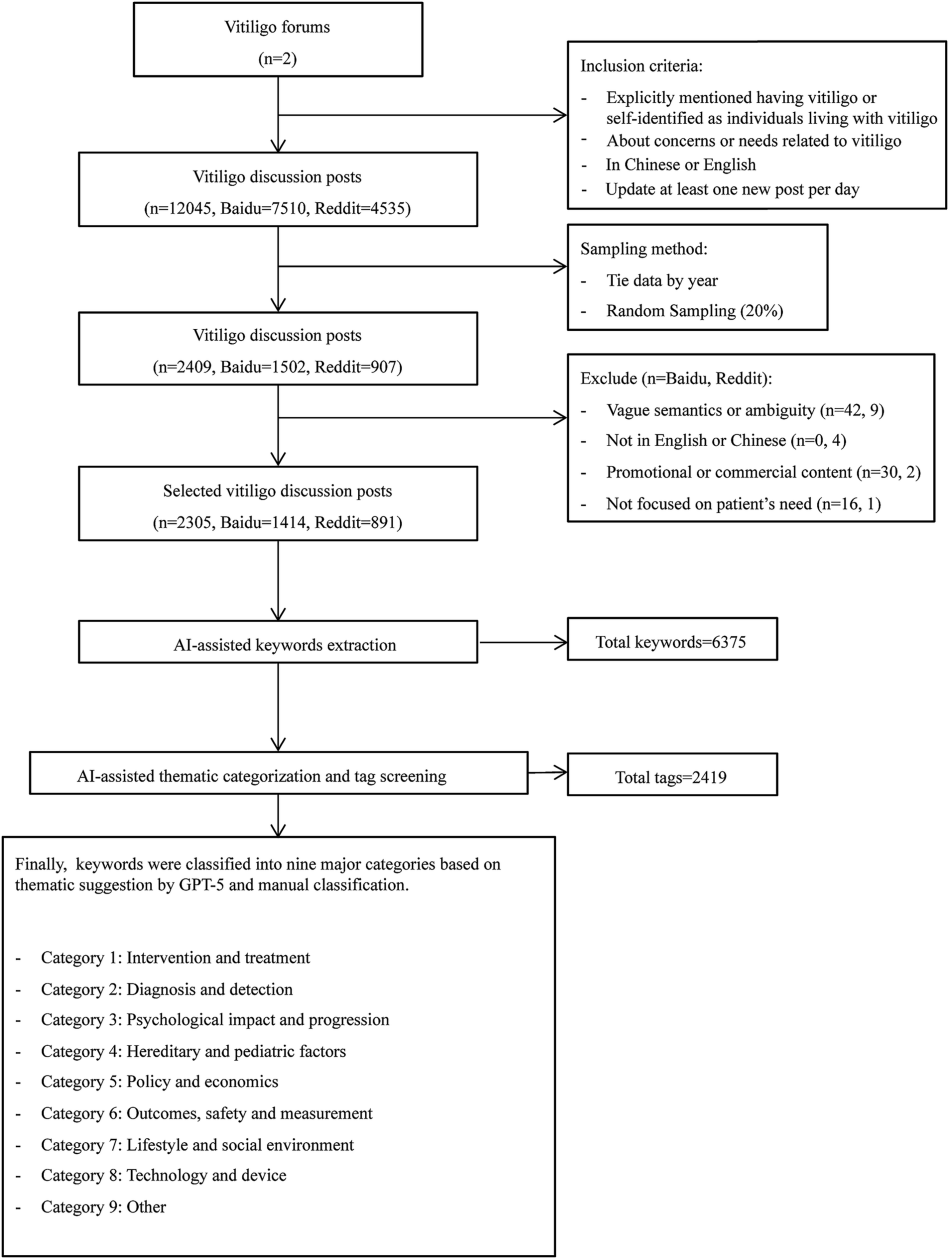
Flowchart of this study.

This study was conducted from September to November 2025. Data were obtained from two prominent open-access forums, Baidu in China and Reddit internationally, which were selected based on the following inclusion criteria (Figure 1): (1) posts that explicitly mentioned having vitiligo or self-identified as individuals living with vitiligo (2) posts containing substantive discussion about concerns or needs related to vitiligo. (3) posts written in Chinese (Baidu) or English (Reddit); and (4) a minimum activity level of one new post per day for the media communities. Relevant communities were identified using keywords including “vitiligo,” “vitiligo diagnosis,” “vitiligo treatment” and “depigmentation.” Two researchers independently managed the retrieval process to encompass all posts published between October 2021 and October 2025, and reached a consensus to include material related to vitiligo needs.

Following data retrieval, all posts were indexed by an independent researcher using unique serial numbers and recorded with their publication dates and source platforms. No usernames, personal identifiers, or direct quotations containing potentially identifiable information were retained in the analytic dataset. To construct a representative dataset while maintaining analytical feasibility, a stratified random sampling strategy was applied based on publication year. Specifically, posts were first grouped by publication year, and approximately 20% of posts from each year were randomly selected using a computer-generated random number procedure to ensure temporal representation across the study period (25, 26). The sampled data was finally screened according to the following criteria: (1) posts with unclear or ambiguous meaning that could not be reliably interpreted; (2) posts written in languages other than English or Chinese; (3) promotional, advertising, or commercial content; and (4) posts not related to vitiligo-related concerns or needs.

### 2.2 AI-assisted keyword extraction and thematic categorization

To analyze unstructured social media text in a structured and reproducible manner, we adopted an AI-assisted workflow for translation and preliminary keyword extraction, followed by researcher-led validation and thematic categorization.

All vitiligo-related posts retrieved from Baidu were translated individually from Chinese into English using GPT-5 with a standardized prompt template. The prompt instructed the model to preserve the original meaning, tone, and contextual details of each post as closely as possible. To minimize potential translation bias, the original Chinese texts were retained throughout the analytic process, and translated posts were independently reviewed by bilingual researchers to assess translation accuracy and semantic consistency.

For both Baidu and Reddit posts, GPT-5 was then used to perform preliminary keyword extraction. The model was instructed to generate two to five concise keywords or short phrases reflecting the main concerns, experience, or informational needs expressed in the text. No predefined keyword dictionary was used at this stage in order to preserve the authenticity and diversity of user-generated expressions.

To ensure consistency and interpretability across platforms and languages, a semi-structured thematic framework was developed prior to formal categorization. GPT-5 was used to provide thematic suggestion for each post based on the extracted keywords. Using a standardized prompt, the model was instructed to recommend the most relevant from the following thematic domain: (1) intervention and treatment; (2) diagnosis and detection; (3) psychological impact and progression; (4) hereditary and pediatric factors; (5) policy and economics; (6) outcomes, safety and measurement; (7) lifestyle and social environment; (8) technology and device; and (9) other. This thematic framework was developed based on prior literature, preliminary familiarization with the dataset, and discussion within the research team before formal categorization (12, 17, 26). Posts and keywords were not forced into predefined categories when a clear conceptual fit was lacking. In such cases, they were assigned to the “other” category, which served as a residual grouping. For ambiguous or borderline cases, categorization was determined through discussion between researchers, with assignment based on the primary expressed concern.

Standardized prompts were applied consistently across all posts, and the prompt templates used for translation, keyword extraction and thematic suggestion are provided in the Supplementary material.

### 2.3 Question extraction and comparative analysis across platforms

To systematically identify the major concerns expressed in social media discussions, we implemented an AI-assisted question extraction and normalization. In this process, GPT-5 was used as a supportive tool for summarizing user concerns and generating standardized question representations from individual posts.

Each post was processed using a standardized prompt instructing the model to summarize the primary concern or informational need expressed in the text as a concise question. For example, posts discussing treatment choices were summarized into standardized questions such as “What treatment method or therapy should I choose?” Similar questions generated by the model were manually consolidated into standardized question formulations in order to reduce redundancy caused by variations in wording. The same prompt template was applied consistently across all posts to ensure procedural consistency. The prompt used for question extraction is provided in the Supplementary material.

After verification, high-frequency questions were identified to represent the major concerns expressed on each platform. Differences in the proportions of high-frequency questions between platforms were evaluated using two-proportion z-tests. *p* values were adjusted using the Benjamini–Hochberg false discovery rate procedure to reduce the risk of false-positive findings. Effect sizes were reported as absolute differences in proportions with corresponding 95% confidence intervals. All tests were two-sided, and adjusted *p* values < 0.05 were considered statistically significant.

### 2.4 Human validation and reliability assessment of AI-assisted outputs

Because GPT-based tools were used in several key analytic steps, we implemented a structured validation framework to assess the reliability, semantic consistency, and reproducibility of AI-assisted outputs.

Two trained reviewers independently evaluated AI-assisted outputs using predefined validation criteria. For Baidu posts, translation adequacy was assessed by comparing the original Chinese text with the English translation. A translation was considered adequate if it preserved the original semantic meaning, including disease-related descriptions, negation, uncertainty, emotional tone, and contextual information. Keyword relevance was defined as whether the extracted keywords were explicitly or implicitly supported by the original post and reflected the user’s main concern. Thematic classification validity was assessed by determining whether the assigned thematic category was consistent with the extracted keywords. Standardized question validity was defined as whether the generated question accurately preserved the user’s original concern without adding unsupported medical interpretation or excessive generalization.

Each AI-assisted output was classified as accepted without revision, accepted after minor revision, or requiring major revision. Minor revisions included changes to wording, clarification of colloquial expressions, or correction of overly broad phrasing that did not alter the original meaning. Major revisions were defined as cases in which the AI-generated output changed the meaning of the original post, introduced unsupported information, omitted the main concern, or assigned an inappropriate thematic category.

Inter-rater agreement between the two reviewers was assessed using Cohen’s kappa for categorical judgments. For ordinal acceptability ratings, weighted Cohen’s kappa was calculated when appropriate. Percentage agreement was also reported to indicate the proportion of outputs accepted without modification or after minor revision. Disagreements between reviewers were resolved through discussion. Cases that could not be resolved by consensus were adjudicated by a senior reviewer with clinical dermatology expertise. The final consensus dataset was used for all subsequent thematic and comparative analyses.

## 3 Results

### 3.1 Basic sample characteristics

The raw dataset included 12,045 posts. After 20% random sampling by year, 2,409 posts were initially included. Prior to final inclusion, 104 posts were excluded due to vague semantics or ambiguity (*n* = 51), promotional or commercial content (*n* = 32), lack of relevance to vitiligo-related needs (*n* = 17), or use of languages other than Chinese or English (*n* = 4). The final dataset for analysis comprised 2,305 vitiligo-related social media posts, published between October 2021 and October 2025 (Baidu: *n* = 1,414; Reddit: *n* = 891). The study selection process is shown in Figure 1.

Keyword extraction yielded 6,375 keyword instances, which were normalized into 2,419 unique terms (“tags”). These keywords were subsequently grouped into nine thematic categories.

**Figure 2:**
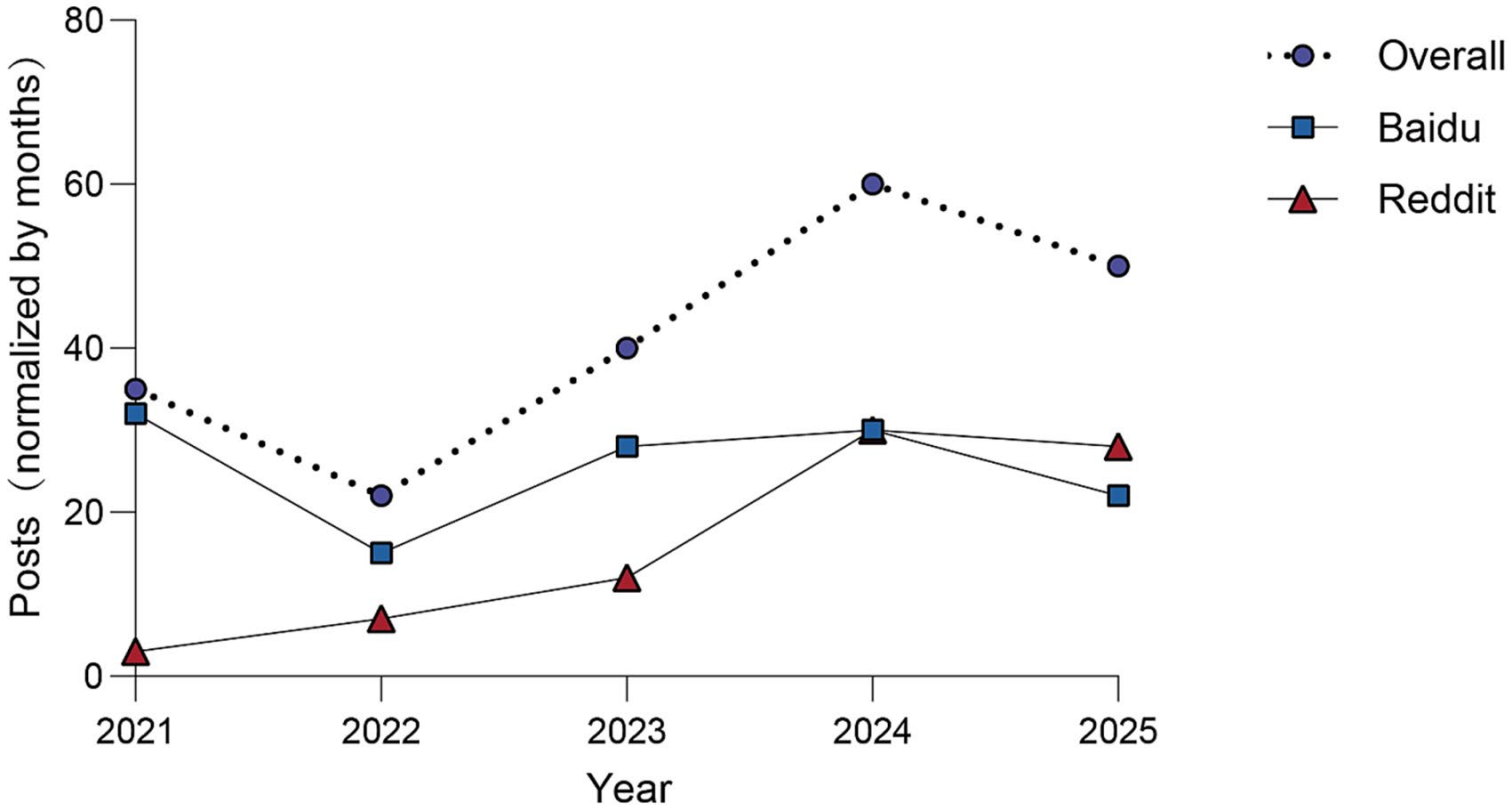
Trends normalized per month in post volume and combined total across two platforms (2021–2025).

**Figure 3:**
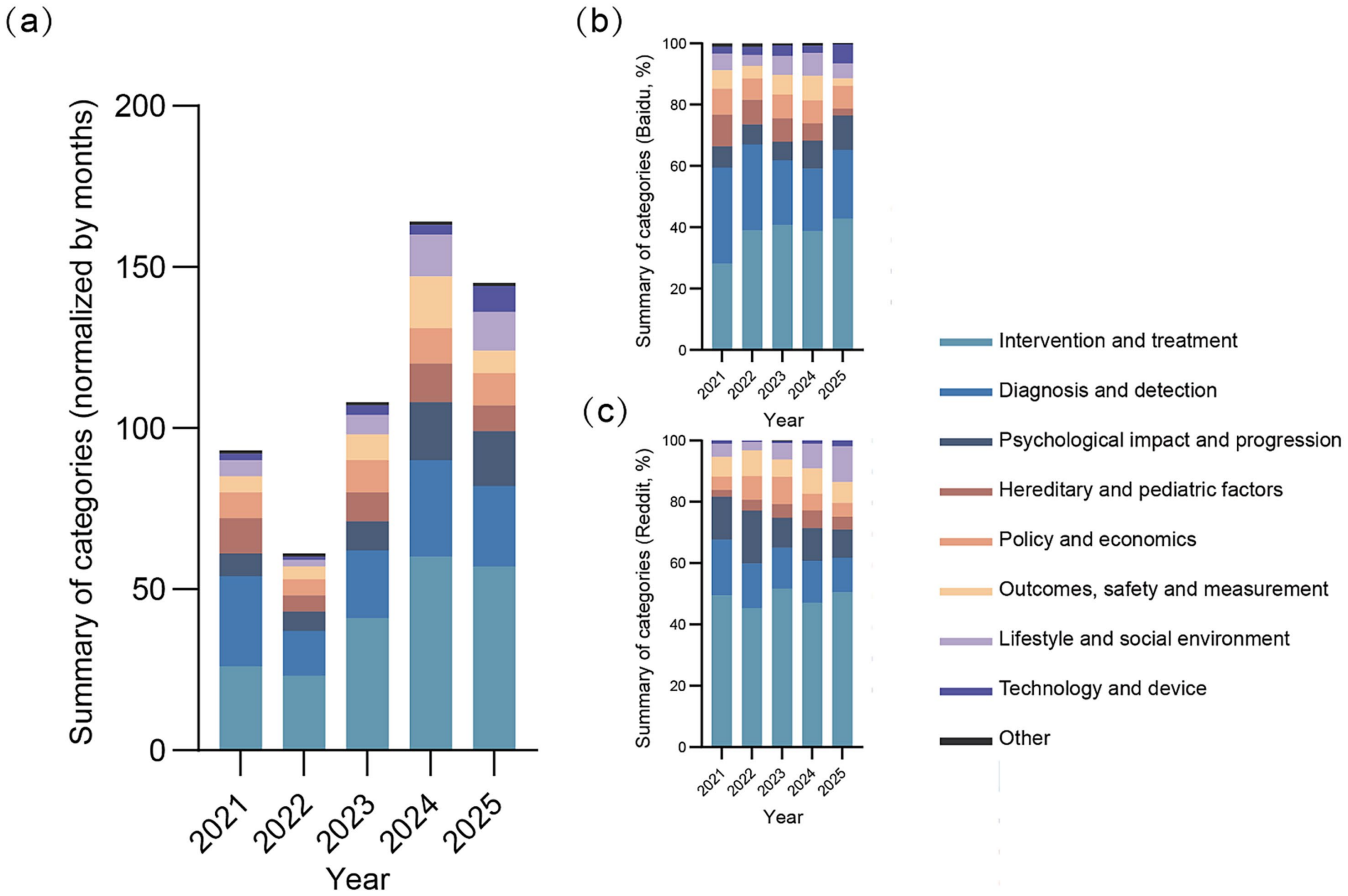
**(a)** Temporal trends normalized per month in total post volume across categories. **(b)** Temporal trends in post proportion by category in Baidu. **(c)** Temporal trends in post proportion by category in Reddit.

The temporal distribution of the discussions was presented in Figure 2 and category-specific longitudinal trends from 2021 to 2025 were presented in Figure 3a. Among the nine categories, Intervention and treatment emerged as the predominant category, exhibiting a consistent year-over-year increase in volume from 2021 to 2024. Conversely, diagnosis and detection and hereditary and pediatric factors maintained relatively stable annual volumes, showed the lowest frequency in 2022, while the remaining categories reached their highest frequency in 2024.

Platform-specific differences in thematic distribution were illustrated in Figures 3b,c. Compared with Reddit, Baidu contained a higher proportion of posts related to diagnosis and detection as well as hereditary and pediatric factors. In contrast, Reddit showed a greater representation of discussions related to psychological impact and progression and to outcomes, safety, and measurement. Discussions related to policy and economics were relatively stable on Baidu but fluctuated on Reddit, with the highest proportion observed in 2023. In addition, lifestyle and social environment showed a gradual increase on Reddit from 2022 to 2025, whereas technology and device reached its highest level on both platforms in 2025.

### 3.2 Distribution of major vitiligo-related concerns

Across all extracted keyword instances, intervention and treatment was the most frequently represented category (2,311/6375, 36.3%), followed by diagnosis and detection (1,330/6375, 20.9%), psychological impact and progression (635/6375, 10.0%), and hereditary and pediatric factors (494/6375, 7.7%) (Table 1).

**Table 1:** Nine thematic categories of vitiligo based on the keywords.

Category (*n*, %) (*N* = 6375)	Word frequency analysis (*n*, %) (*N* = 6,375)	
Intervention and treatment (2311, 36.3%)	General treatment method (212, 3.3%)	Repigmentation efficiency (85, 1.3%)
	Opzelura (95, 1.5%)	Cosmetic camouflage (64, 1%)
Diagnosis and detection (1,330, 20.9%)	Differential diagnosis (245, 3.9%)	Device selection (37, 0.6%)
	Disease diagnosis (117, 1.8%)	Adverse effects (33, 0.5%)
Psychological impact and progression (635, 10.0%)	Disease progression (46, 0.72%)	Long-Standing disease (18, 0.28%)
	Psychological distress (25, 0.39%)	Progressive symptoms (18, 0.28%)
Hereditary and pediatric factors (494, 7.7%)	Pediatric case (29, 0.45%)	Epidemiology (10, 0.16%)
	Pediatric onset (11, 0.17%)	Patient experience (10, 0.16%)
Policy and economics (487, 7.6%)	Care navigation (55, 0.86%)	Treatment access (20, 0.31%)
	Hospital recommendation (37, 0.58%)	Hospital evaluation (15, 0.24%)
Outcomes, safety and measurement (450, 7.1%)	Efficacy inquiry (20, 0.31%)	Treatment outcome (19, 0.3%)
	Safety considerations (20, 0.31%)	Curability question (16, 0.25%)
Lifestyle and social environment (435, 6.8%)	Peer support (77, 1.21%)	Psychological support (20, 0.31%)
	Sun exposure (41, 0.64%)	Dietary management (15, 0.24%)
Technology and device (183, 2.9%)	Home phototherapy device (22, 0.35%)	Home UVB device (12, 0.19%)
	308 nm phototherapy (15, 0.24%)	Device recommendation (12, 0.19%)
Other (50, 0.8%)	Early-Stage vitiligo (8, 0.13%)	Mild vitiligo (3, 0.05%)
	Chronic lesion (3, 0.05%)	Acral vitiligo (2, 0.03%)

Within the intervention and treatment category, common topics included general treatment methods (212/6375, 3.3%), Opzelura (95/6375, 1.5%), repigmentation efficacy (85/6375, 1.3%), and cosmetic camouflage (64/6375, 1.0%) (Table 1). Frequently represented treatment-related questions included “What treatment method or therapy should I choose?” (794/2305, 34.45%), “Is the treatment effective, and what is the risk of relapse or the overall prognosis?” (196/2305, 8.50%), and “What is the correct dosage and how long should I take it?” (102/2305, 4.43%) (Table 2). Posts in this category also frequently referred to combination therapy, treatment response, and medication safety.

**Table 2:** High-frequency standardized questions in vitiligo-related discussions.

No.	Questions	Frequency (*n*, %) (*N* = 2,305)
1	What is the main issue or diagnosis regarding my specific skin condition?	(811, 35.18%)
2	What treatment method or therapy should I choose?	(794, 34.45%)
3	How can I book an appointment, and which doctor or hospital should I choose?	(262, 11.37%)
4	Is the treatment effective, and what is the risk of relapse or the overall prognosis?	(196, 8.5%)
5	What side effects or safety risks should I be aware of for this medication?	(160, 6.94%)
6	Is this treatment suitable for pregnant women, children, or older adults?	(107, 4.64%)
7	What is the correct dosage and how long should I take it?	(102, 4.43%)
8	How can I manage anxiety, depression, or other psychological stress?	(75, 3.25%)
9	How much will it cost, and can it be reimbursed by insurance?	(72, 3.12%)
10	Which diagnostic method should I take, and how should I interpret the results?	(66, 2.86%)
11	What lifestyle, diet, or exercise changes should I follow?	(63, 2.73%)
12	What do current guidelines or policies recommend, and what are the indications or contraindications?	(57, 2.47%)
13	Where can I obtain the treatment or medicine, and is it currently available?	(48, 2.08%)
14	Should I get vaccinated or take preventive measures?	(40, 1.74%)

The diagnosis and detection category were the second most frequent theme. Common keywords within this domain included differential diagnosis (245/6375, 3.9%) and disease diagnosis (117/6375, 1.8%) (Table 1). The most frequent standardized question overall was “What is the main issue or diagnosis regarding my specific skin condition?” (811/2305, 35.18%) (Table 2). Additional concerns in this category involved diagnostic testing, interpretation of examination results, and device selection.

The psychological impact and progression category included concerns related to disease progression, long-standing disease, psychological distress, stigma, and self-esteem. Representative posts referred to worsening lesions during treatment, anxiety about disease progression, and distress related to social interactions. Discussions in this category also included concerns about marriage and romantic relationships among individuals affected by vitiligo.

Hereditary factors and pediatric considerations comprised the fourth category, representing 7.7% of discussions. Within this domain, online discourse commonly expressed concerns regarding incidence rates, age of onset, disease progression, and prognosis in pediatric populations, as well as the probability of vertical transmission from parents to offspring. Frequently posed questions reflected these concerns: “Could this presentation indicate vitiligo in my child? Could someone provide assessment and recommendations?” and “What is the heritability of vitiligo? Is hereditary vitiligo associated with greater treatment resistance?”

Beyond the four principal categories aforementioned, vitiligo-related concerns additionally encompassed financial burden, medication accessibility and adverse effects, the influence of lifestyle and environmental factors on vitiligo pathogenesis, healthcare facility recommendations, and home-based phototherapeutic device utilization. Furthermore, online discourse expressed interest in the potential association between COVID-19 vaccination and vitiligo induction. A word cloud visualization was generated to synthesize these diverse topics and provide a comprehensive overview of online discussions (Supplementary material).

### 3.3 Comparative analysis across platforms

Detailed cross-platform comparisons of the 10 high-frequency questions are presented in Table 3. Among these questions, six were more frequently represented on Baidu, whereas four were more frequently represented on Reddit.

**Table 3:** Differences in patient-expressed concerns between Baidu and Reddit.

No.	Questions	*n* = (Baidu, Reddit) *N* = (1,414, 891)	Difference in proportion (95% CI)	*P* ^a^
1	Am I contagious, and how should I prevent transmission?	(9, 0)	0.64 (0.22, 1.05)	*
2	How can I book an appointment, and which doctor or hospital should I choose?	(230, 32)	12.7 (10.0, 14.9)	***
3	How can I manage anxiety, depression, or other psychological stress?	(63, 12)	3.2 (1.79, 4.42)	***
4	Is the treatment effective, and what is the risk of relapse or the prognosis?	(147, 49)	4.5 (2.71, 7.08)	***
5	Is this treatment suitable for pregnant women, children, or older adults?	(80, 27)	2.7 (0.98, 4.28)	**
6	Should I get vaccinated or take preventive measures?	(7, 33)	1.5 (0.57, 2.53)	**
7	What is the correct dosage and how long should I take it?	(50, 52)	−2.3 (−4.1, −0.48)	**
8	What side effects or safety risks should I be aware of for this medication?	(55, 105)	−7.9 (−10.2, −5.6)	***
9	Where can I obtain the treatment or medicine, and is it currently available?	(15, 33)	−2.6 (−4.0, −1.3)	***
10	Which diagnostic tests should I take, and how should I interpret the results?	(30, 36)	−1.9 (−3.1, −0.4)	**

^a^P adjusted by Benjamini–Hochberg correction. * adjusted *P* < 0.05, ** adjusted *P* < 0.01, *** adjusted *P* < 0.001.

Compared with Reddit, Baidu showed higher proportions of posts asking about healthcare provider or hospital selection (230 vs. 32; difference in proportions 12.7, 95% CI 10.0–14.9%; adjusted *p* < 0.001), treatment efficacy and prognosis (147 vs. 49; 4.5, 95% CI 2.71–7.08%; adjusted *p* < 0.001), psychological stress management (63 vs. 12; 3.2, 95% CI 1.79–4.42%; adjusted *p* < 0.001), treatment suitability for pregnant women, children, or older adults (80 vs. 27; 2.7, 95% CI 0.98–4.28%; adjusted *p* < 0.01), vaccination or preventive measures (33 vs. 7; 1.5, 95% CI 0.57–2.53%; adjusted *p* < 0.01), and disease transmissibility or prevention (9 vs. 0; 0.64, 95% CI 0.22–1.05%; adjusted *p* < 0.05).

In contrast, Reddit showed higher proportions of posts asking about medication side effects or safety risks (55 vs. 105; difference in proportions −7.9, 95% CI -10.2% to −5.6%; adjusted *p* < 0.001), treatment availability or access (15 vs. 33; −2.6, 95% CI -4.0% to −1.3%; adjusted *p* < 0.001), medication dosage and treatment duration (50 vs. 52; −2.3, 95% CI -4.1% to −0.48%; adjusted *p* < 0.01), and interpretation of diagnostic tests (30 vs. 36; −1.9, 95% CI -3.1% to −0.4%; adjusted *p* < 0.01).

The largest cross-platform difference was observed for questions related to healthcare provider or hospital selection, which were substantially more common on Baidu. By contrast, the largest Reddit-overrepresented topic concerned medication side effects and safety risks.

Quantitative validation results were shown in Supplementary Tables S1, S2.

## 4 Discussion

This study used an AI-assisted analytical workflow to examine major concerns expressed in vitiligo-related discussions on Baidu and Reddit, providing a real-world cross-platform view of informational and practical needs. Overall, treatment-related concerns were the most prominent shared topic across both platforms, while substantial differences were observed in the distribution of high-frequency standardized questions. Compared with Reddit, Baidu discussions more often focused on diagnosis, healthcare provider selection, prognosis, and concerns involving specific populations such as children, pregnant women, and older adults. In contrast, Reddit discussions more frequently emphasized medication safety, dosage, treatment access, and interpretation of diagnostic tests. Together, these findings suggest that social media discussions may provide a supplementary perspective on concerns related to vitiligo that may not be fully captured in conventional clinical encounters.

A major finding of this study was that treatment-related concerns dominated discussions on both platforms. This aligns with findings by Teasdale et al. (27) and Ezzedine et al. (13), confirming that vitiligo patients’ primary objective remains achieving rapid and durable repigmentation. Across categories, intervention and treatment represented the largest proportion and several of the most frequent standardized questions were directly related to treatment selection, therapeutic efficacy, relapse risk, dosage, and safety. This pattern is consistent with the chronic and visible nature of vitiligo, in which uncertainty regarding repigmentation, maintenance of treatment response, and long-term disease control remains a central concern (28–31). The prominence of these treatment-oriented questions suggests that many users turn to social media not only for general information, but also for practical guidance. As treatment regimens continue to evolve, patients are faced with a multitude of therapeutic options. In addition to traditional treatments such as topical corticosteroids and phototherapy, novel interventions like topical JAK inhibitors (e.g., ruxolitinib cream, brand name Opzelura) have entered public awareness (4, 29–32). The high frequency of keywords like “Opzelura” highlights this trend. Given the information asymmetry between healthcare providers and patients, it is becoming increasingly necessary for patients to obtain key insights into real-world drug efficacy from social media platforms from their own perspective (17, 33–35). This phenomenon may also reflect the potential importance of long-term collaboration and shared decision-making in the chronic disease management of vitiligo. (36, 37).

At the same time, the Reddit appeared to differ in the specific dimensions of treatment-related concerns that were most salient. On Reddit, questions about medication side effects, dosage, treatment duration, and access to therapies were more frequently represented. These findings may reflect a stronger focus on treatment management and safety monitoring in English-language discussions, where users often seek highly specific, experience-based advice. The prominence of questions about adverse effects is particularly important, because concern about treatment-related harm may contribute to hesitation, poor adherence, or early discontinuation (38). In chronic dermatologic conditions, adherence is often influenced not only by perceived efficacy but also by perceived treatment burden and safety (12, 39). Our findings may highlight the potential relevance of transparent clinician-patient communication regarding expected benefits, adverse effects, duration of therapy, and strategies for managing uncertainty during treatment.

Baidu discussions more frequently involved diagnosis, healthcare provider or hospital selection, prognosis, and questions related to special populations. The largest cross-platform difference was observed for questions about how to book appointments and which doctor or hospital to choose, suggesting that healthcare navigation is a particularly prominent concern in the Chinese-language online environment (40, 41). Questions about differential diagnosis, disease confirmation, and interpretation of disease status were also common. These findings may indicate that, for many users, uncertainty begins before treatment itself—at the level of recognizing the condition, confirming the diagnosis, and identifying a reliable pathway to specialist care (42, 43). Moreover, questions concerning pregnant women, children, and older adults were more frequently represented on Baidu, suggesting that family-centered decision-making and concern for vulnerable populations may play a particularly important role in these discussions (10, 12, 44, 45). Although the underlying causes of these differences cannot be determined from the present data, they may reflect variation in health system navigation, online help-seeking practices, and sociocultural expectations across platforms.

Beyond treatment and diagnosis, this study also highlighted psychosocial concerns that are often underrepresented. Posts categorized under psychological impact and progression frequently referred to anxiety, stigma, self-esteem, worsening lesions, and uncertainty about long-term disease course. Importantly, discussions also included concerns related to marriage and romantic relationships, especially on the Chinese platform. These posts suggest that the burden of vitiligo extends beyond symptom management and may affect social participation, intimate relationships, and future life planning (11, 46, 47). Thompson et al.’s work on Asian populations (48) similarly demonstrated that cultural emphasis on skin appearance intensifies patient shame. Such findings reinforce the view that vitiligo should not be understood solely as a pigmentary disorder, but also as a condition with important psychosocial and relational consequences (10, 13). They may further suggest that emotional well-being, stigma reduction, and social functioning are important aspects that could be considered alongside medical treatment.

The study also identified concerns related to heredity, childhood onset, and disease course in children, which further broaden the scope of unmet needs reflected in online discussions. Questions about whether a child’s skin changes could represent vitiligo, whether the disease is hereditary, and whether inherited disease may be more difficult to treat point to the central role of uncertainty in family contexts (44, 45). These concerns are relevant from a public health perspective, as they suggest that health communication related to vitiligo should not be limited to treatment options alone. Clear, accessible, and evidence-based communication is also needed regarding diagnosis, inheritance, pediatric care, prognosis, and support for caregivers and families.

Another important implication of this study relates to the role of social media as both a source of support and a potential source of misinformation. Although online platforms provide accessible spaces for information exchange, peer support, and experiential learning, they also facilitate the circulation of non-evidence-based advice, including unverified remedies, overly restrictive dietary recommendations, and potentially misleading treatment narratives (49, 50). This creates a dual challenge: users increasingly rely on online communities for health-related guidance, yet the quality and accuracy of that guidance may vary substantially. Our findings may suggest a potential role for stronger professional engagement in digital health communication.

At the same time, the findings should be interpreted with caution. First, because this study relied on publicly available social media posts, the clinical identities and diagnostic status of users could not be independently verified. Although we restricted inclusion to posts that explicitly self-identified vitiligo, this approach does not ensure clinical confirmation. Therefore, the findings should be interpreted as reflecting concerns expressed in vitiligo-related online discourse rather than validated patient-reported outcomes from clinically confirmed populations. Second, the sampling proportion was selected pragmatically rather than based on a formal statistical calculation. Although stratified random sampling was used to improve temporal representativeness, this approach may have underrepresented infrequent but potentially clinically relevant topics. This may be especially relevant for culturally specific expressions, colloquial language, or context-dependent medical terminology. Third, although keyword level thematic distribution allows for a more granular representation of concerns, may give greater weight to longer or more complex posts. Fourth, high-frequency questions may not capture all issues that are clinically important, especially those expressed less often but carrying substantial emotional or practical significance, and predefined thematic framework may influence how concerns are categorized. Fifth, Baidu and Reddit differ not only in language, but also in their institutional and sociocultural contexts. For example, differences in healthcare access, referral systems, and patterns of specialist consultation may influence the types of questions users raise online. Cultural norms may also shape whether concerns are expressed in terms of family responsibility, stigma, treatment risk, or individual self-management. In addition, the demographic composition of users on the two platforms is likely not equivalent, and platform-specific features—such as anonymity, moderation practices, and algorithmic visibility—may further affect what kinds of posts are created or retained. Finally, although efforts were made to standardize prompts and processing steps, AI-assisted methods may introduce variability due to model updates or inherent stochasticity.

The use of publicly available social media data in health research raises important ethical considerations. Although such data are accessible without restriction, users may not fully anticipate that their posts will be systematically analyzed for research purposes, particularly when discussing sensitive health-related experiences (51, 52). This has led to ongoing debates regarding privacy, informed consent, and the potential for re-identification in digital health research (51, 52). In this study, efforts were made to minimize these risks by avoiding the collection of identifiable information, analyzing data in aggregated form, and refraining from reporting verbatim content. However, ethical considerations in this field extend beyond formal regulatory requirements. Researchers must also consider contextual integrity, user expectations, and the responsible interpretation of online discourse.

Despite these limitations, the present study provides a cross-platform, data-driven perspective on vitiligo-related concerns as expressed in real-world online discussions. The findings suggest that social media can reveal practical, emotional, and informational needs that extend beyond conventional biomedical outcomes, including concerns about treatment safety, diagnosis, healthcare navigation, stigma, family burden, and relationship anxiety. These results may provide exploratory insights relevant to patient-centered care, digital health communication, and public health education. Future research could build on this work by integrating social media analysis with qualitative interviews, surveys, or clinically characterized cohorts. In addition, incorporating more advanced natural language processing techniques—such as topic modeling, semantic embeddings, clustering methods, or large language model–based representations—may enable a deeper exploration of latent thematic structures, contextual relationships, and cross-platform semantic patterns. Additionally, more inductive approaches, such as topic modeling or embedding-based clustering, to identify latent thematic structures and complement structured categorization frameworks should be explored.

## 5 Conclusion

In conclusion, this AI-assisted cross-platform study showed that vitiligo-related social media discussions on Baidu and Reddit were dominated by treatment-related concerns, while also revealing clear differences in the distribution of high-frequency questions across platforms. Beyond treatment and diagnosis, the findings highlighted substantial psychosocial, family-related, and practical concerns that may not be fully captured in routine clinical encounters. These results suggest that social media discussions may provide a supplementary perspective on concerns related to vitiligo. While the findings are descriptive and based on indirect observational data, they may offer exploratory insights that could inform future research, patient education, and digital health communication strategies.

## Data Availability

The original contributions presented in the study are included in the article/Supplementary material, further inquiries can be directed to the corresponding author/s.
