# Supplementary material for: Understanding vitiligo-related concerns through online discourse: an AI-assisted cross-platform study of Chinese and global communities
Source: Front Public Health. 2026 May 13;14:1832699. doi: 10.3389/fpubh.2026.1832699 (PMC13212291; doi:10.3389/fpubh.2026.1832699)
Supplement: Supplementary file 1 [file Supplementary_file_1.docx]

Supplementary Material

# Supplementary Data

**Prompt 1: Translation of Chinese posts**

Please translate the following Chinese social media post into English.

Requirements:

1. Preserve the original meaning, tone, and contextual information as accurately as possible.
2. Do not summarize or omit any information.
3. Do not add interpretation or new content that is not present in the original text.
4. Maintain the structure and intent of the original post.

Output only the English translation.

**Prompt 2: Keyword extraction**

Please read the following social media post related to vitiligo and extract 2 to 5 concise keywords or short phrases that best represent the main concern, experience, or informational need expressed in the text.

Requirements:

1. Keywords should reflect the main topic or concern expressed in the post.
2. Avoid overly general terms (e.g., “health,” “problem”).
3. Use short phrases if necessary to capture the meaning accurately.
4. Do not infer information that is not clearly expressed in the text.

Output only the keywords as a list

**Prompt 3: Thematic suggestion**

Based on the following keyword(s), suggest which thematic domain the post may belong to among the following categories:

1. Intervention and treatment
2. Diagnosis and detection
3. Psychological impact and progression
4. Hereditary and pediatric factors
5. Policy and economics
6. Outcomes, safety and measurement
7. Lifestyle and social environment
8. Technology and device
9. Other

Provide only the most relevant category suggestion.

**Prompt 4: Question Extraction and Standardization**

Please read the following social media post related to vitiligo and identify the single dominant concern or informational need expressed by the user. Rewrite this concern as one concise standardized question suitable for analytical comparison across posts.

Instructions:

Identify only the most central concern expressed in the post.

1. If multiple concerns appear, select the dominant one.
2. Rewrite it as a short, grammatically complete question in English.
3. Preserve the original meaning as closely as possible.
4. Do not introduce information not explicitly stated in the post.
5. Use neutral and standardized wording to allow comparison across posts.

For Example: posts discussing treatment choices may be standardized into questions such as “What treatment method or therapy should I choose?”

# Supplementary Figure

**Figure S1** Word Cloud of Keyword Frequency Distribution Across Two Platforms.


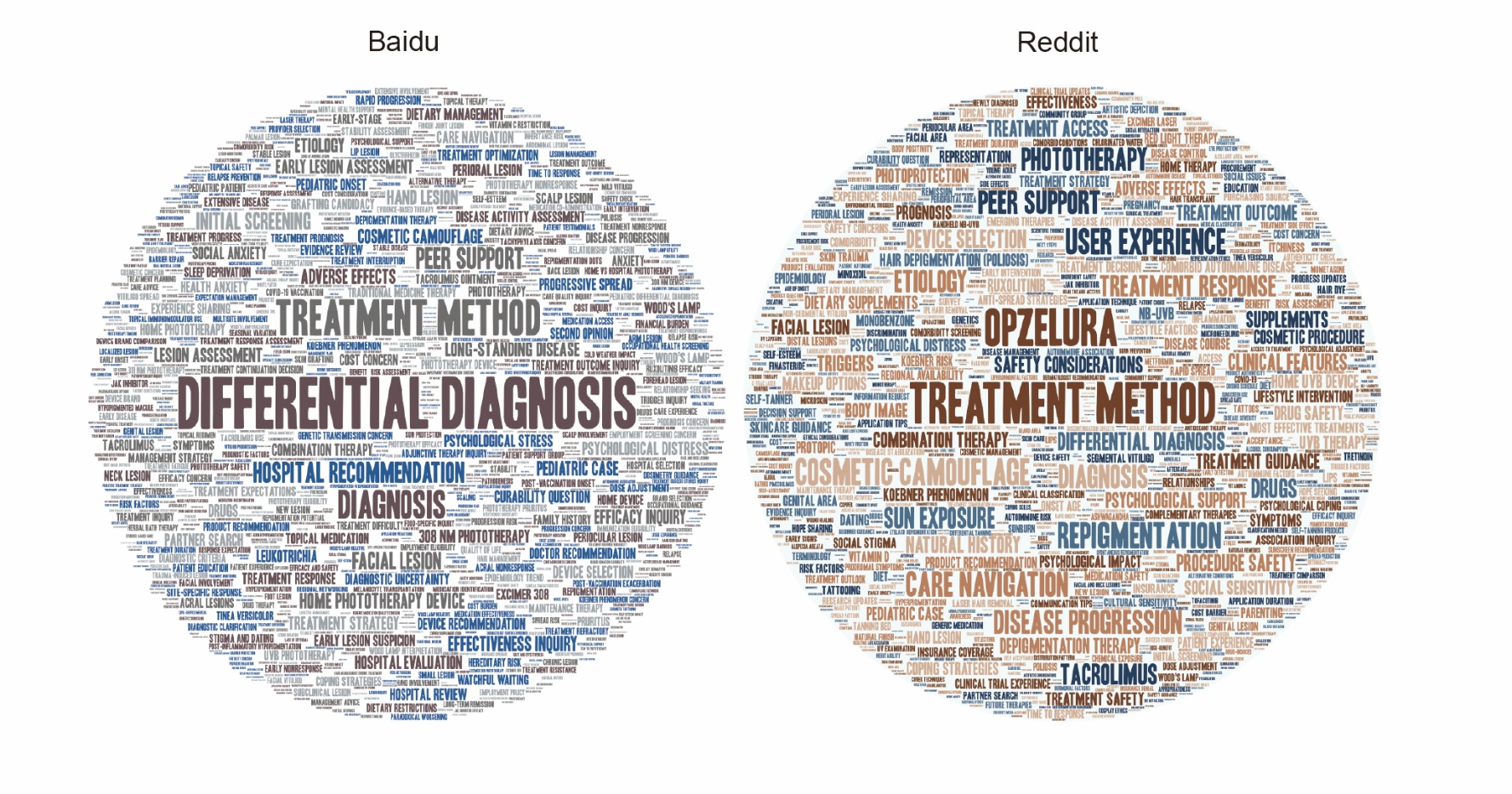


# Supplementary Table

**Supplementary Table S1. Full-sample validation outcomes of AI-assisted outputs**

| Platform | Unit of validation | Units assessed | Without revision, n (%) | Minor revision, n (%) | Major revision, n (%) | Main reasons for revision |
| --- | --- | --- | --- | --- | --- | --- |
| Baidu | Post-level translation | 1,414 | 1,378 (97.5) | 36 (2.5) | 0 | ambiguous symptom descriptions; emotional tone not fully preserved. |
| Reddit | Not applicable | - | - | - | - | - |
| Baidu | Keyword set per post | 1,414 | 1,291(91.3) | 98 (6.9) | 25 (1.8) | Overly broad keyword;  missed main concern;  redundant terms. |
| Reddit | Keyword set per post | 891 | 762 (85.5) | 109 (12.2) | 20 (2.2) |  |
| Overall | Keyword set per post | 2,305 | 2053 (89.1) | 207 (9.0) | 41 (1.8) |  |
| Baidu | Post-level standardized question | 1,414 | 1,076 (76.1) | 234 (16.4) | 104 (7.4) | Unsupported inference;  redundant wording;  incomplete preservation of user intent;  changed the meaning of the original post;  introduced unsupported information. |
| Reddit | Post-level standardized question | 891 | 718 (80.6) | 100 (11.2) | 73 (8.2) |  |
| Overall | Post-level standardized question | 2,305 | 1,794 (77.8) | 334 (14.5) | 177 (7.7) |  |
| Baidu | Keywords-level thematic category | 3,892 | 3,349 (86.0) | - | 543 (14.0) | Incorrect category |
| Reddit | Keywords-level thematic category | 2,483 | 2,198 (88.5) | - | 285 (11.5) |  |
| Overall | Keywords-level thematic category | 6,375 | 5,547 (87.0) | - | 828 (13.0) |  |

**Note.** Minor revisions were defined as wording corrections, clarification of colloquial expressions, removal of redundant terms, or minor adjustments that did not alter the original meaning. Major revisions were defined as cases in which the AI-generated output changed the meaning of the original post, introduced unsupported information, omitted the main concern, or assigned an inappropriate thematic category. For keywords-level thematic category evaluation, minor revisions were not applicable because such outputs do not involve wording or semantic preservation; therefore, only major revisions were assessed.

**Supplementary Table S2. Inter-rater agreement for validation of AI-assisted outputs**

| Validation domain | Platform | Unit of assessment | No. assessed | Rating scale | Cohen’s kappa or weighted kappa | 95% CI | Interpretation |
| --- | --- | --- | --- | --- | --- | --- | --- |
| Translation adequacy | Baidu | Post-level translation | 1,414 | Adequate/ inadequate | 0.627 | (0.503, 0.751) | Substantial |
| Keyword relevance | Baidu | Keyword set per post | 1,414 | Accepted/minor revision/major revision | 0.664 (weighted) | (0.603, 0.725) | Substantial |
| Keyword relevance | Reddit | Keyword set per post | 891 | Accepted/minor revision/major revision | 0.842 (weighted) | (0.795, 0.889) | Excellent |
| Keyword relevance | Overall | Keyword set per post | 2,305 | Accepted/minor revision/major revision | 0.689 (weighted) | (0.646, 0.732) | Substantial |
| Standardized question validity | Baidu | Post-level standardized question | 1,414 | Accepted/minor revision/major revision | 0.881 (weighted) | (0.842, 0.920) | Excellent |
| Standardized question validity | Reddit | Post-level standardized question | 891 | Accepted/minor revision/major revision | 0.877 (weighted) | (0.834, 0.920) | Excellent |
| Standardized question validity | Overall | Post-level standardized question | 2,305 | Accepted/minor revision/major revision | 0.800 (weighted) | (0.761, 0.831) | Substantial |
| Thematic classification | Baidu | Nine-category thematic assignment | 3,892 | Correct/incorrect | 0.682 | (0.658, 0.706) | Substantial |
| Thematic classification | Reddit | Nine-category thematic assignment | 2,483 | Correct/incorrect | 0.655 | (0.613, 0.697) | Substantial |
| Thematic classification | Overall | Nine-category thematic assignment | 6,375 | Correct/incorrect | 0.673 | (0.653, 0.693) | Substantial |

**Note.** Cohen’s kappa was used for nominal categorical ratings, including translation adequacy and thematic classification. Weighted Cohen’s kappa was used for ordinal acceptability ratings, including keyword relevance and standardized question validity, when outputs were classified as accepted without revision, accepted after minor revision, or requiring major revision. Interpretation of kappa values followed conventional thresholds: ≤0.20, slight agreement; 0.21–0.40, fair agreement; 0.41–0.60, moderate agreement; 0.61–0.80, substantial agreement; and 0.81–1.00, excellent agreement.
